# Towards the development of multifunctional molecular indicators combining soil biogeochemical and microbiological variables to predict the ecological integrity of silvicultural practices

**DOI:** 10.1111/1751-7915.12348

**Published:** 2016-02-08

**Authors:** Vincent Peck, Liliana Quiza, Jean‐Philippe Buffet, Mondher Khdhiri, Audrey‐Anne Durand, Alain Paquette, Nelson Thiffault, Christian Messier, Nadyre Beaulieu, Claude Guertin, Philippe Constant

**Affiliations:** ^1^ INRS‐Institut Armand‐Frappier531 boulevard des PrairiesLavalQuébecCanadaH7V 1B7; ^2^ Centre d’étude de la forêtUniversité du Québec à MontréalCase postale 8888, succursale Centre‐villeMontréalQuébecCanadaH3C 3P8; ^3^ Direction de la recherche forestièreMinistère des Forêts, de la Faune et des Parcs2700 EinsteinQuébecQuébecCanadaG1P 3W8; ^4^ Institut des Sciences de la Forêt Tempérée (ISFORT)Université du Québec en Outaouais (UQO)58 rue PrincipaleRiponQuébecCanadaJ0V 1V0; ^5^ Produits Forestiers Résolu2419 Route 155 sudLa TuqueQuébecCanadaG9X 3N8

## Abstract

The impact of mechanical site preparation (MSP) on soil biogeochemical structure in young larch plantations was investigated. Soil samples were collected in replicated plots comprising simple trenching, double trenching, mounding and inverting site preparation. Unlogged natural mixed forest areas were used as a reference. Analysis of soil nutrients, abundance of bacteria and gas exchanges unveiled no significant difference among the plots. However, inverting site preparation resulted in higher variations of gas exchanges when compared with trenching, mounding and unlogged natural forest. A combination of the biological and physicochemical variables was used to define a multifunctional classification of the soil samples into four distinct groups categorized as a function of their deviation from baseline ecological conditions. According to this classification model, simple trenching was the approach that represented the lowest ecological risk potential at the microsite level. No relationship was observed between MSP method and soil bacterial community structure as assessed by high‐throughput sequencing of bacterial 16S rRNA gene; however, indicator genotypes were identified for each multifunctional soil class. This is the first identification of multifunctional molecular indicators for baseline and disturbed ecological conditions in soil, demonstrating the potential of applied microbial ecology to guide silvicultural practices and ecological risk assessment.

## Introduction

Tree plantations are gaining increased interest to protect natural forests, restore ecosystem services and meet various social needs. For instance, more than 15% of current wood production is supported by tree plantations and this contribution is expected to rise in the future (Paquette and Messier, 2009). Mechanical site preparation (MSP) is a common practice to improve seedling performance in tree plantations dedicated to intensive wood production and forest regeneration. This approach, applied following clear‐cut or variable retention logging, involves the utilization of heavy machineries to break soil structure in order to improve soil physical conditions limiting tree growth and control competing vegetation (Löf *et al*., 2012). Soil scarification and mounding are the most usual MSP methods. Scarification consists in mixing organic and mineral upper layers of soil by trenching. This technique increases soil aeration and temperature, while favouring nutrient availability by accelerating nitrogen mineralization and limiting the invasion of competing vegetation (Prévost, 1992; Thiffault and Jobidon, 2006). Mounding consists in creating elevated planting spots where soil is excavated and deposited on the ground, next to the ditch, while inverting the soil horizons to get mineral soil on the top and an organic layer at the bottom (Sutton, 1993). As an alternative to mounding to increase worker safety for following operations such as cleaning and thinning, excavated soil can also be placed back into the original ditch, resulting in inverting site preparation. Mounding treatments provide the beneficial effects on soil aeration, temperature, competing vegetation control and nutrient availability observed with scarification and are particularly suitable to get seedling spots free from water logging conditions in wet areas (Kabrick *et al*., 2005; Simon *et al*., 2013). Mounding and inversion using an excavator are also expected to be less damaging to the environment than regular trenching, disturbing a lesser proportion of the treated area (especially inversion) and avoiding the creation of linear trenches that may cause soil erosion (Buitrago *et al*., 2015).

Increased soil temperature, followed by the stimulation of organic carbon mineralization and nutrient release are the prevailing soil disturbances caused by MSP (Jandl *et al*., 2007). In contrast to plant diversity, which is expected to be resilient to this management practice (Haeussler *et al*., 2004), the resilience, resistance or vulnerability of soil microbiome to these marked soil disturbances remains unknown. This question is of critical importance in ecosystem‐based forest management where establishment of tree plantations must exert minimal alteration or even restore ecosystem services sustained in natural forests (Martin *et al*., 2014). Because microorganisms play a crucial role in global biogeochemical cycles and closely interact with vegetation through nutrient transfers as well as water retention, the composition of soil microbial communities can be seen as an indicator of soil ecosystem functioning. For instance, high microbial biodiversity was shown to promote soil multiple ecological functions including plant diversity and nutrient cycling (Wagg *et al*., 2014), resistance to the invasion and survival of allochthonous pathogen species (van Elsas *et al*., 2012) as well as resistance and resilience of microbial community structure to certain environmental stress (Tardy *et al*., 2014).

The overarching objective of this study was to compare the metabolic activity and composition of soil bacterial community between recently clear‐cut and site prepared plots within an hybrid larch (*Larix × marschlinsii* Coaz) plantation and unlogged natural mixed boreal forest conservation areas to assess the impact of different MSP on soil ecosystem functioning a few years following treatments. The rationale for this approach is the identification of soil biogeochemical processes and microbes affected by MSP to select silvicultural practices offering the best early environmental performance at the microsite level. We tested the hypothesis that both the conversion of natural forest to a hybrid larch monoculture and the intensification of MSP treatments reduce the activity and the taxonomic diversity of soil bacterial community in the early years after this conversion. We finally combined soil biogeochemical and microbiological data sets to explore the relevance of microbial molecular indicators to predict the environmental impact and guide silviculture practices. In contrast to previous investigations comparing single parameter such as soil enzyme activities between reference sites and managed forests (*e.g*. Staddon *et al*., 1999), we have combined multiple biogeochemical variables as a metric to classify soil under an original multifunctional system and searched for indicator 16S rRNA gene sequences restricted to specific soil multifunctional classes. Biogeochemical variables were selected to get a broad classification system, including gaseous exchange involving taxonomically diverse and specialized microbial guilds. Under this framework, we expected to identify indicators for baseline or disturbed ecological conditions in soil and to explore the potential of soil bacterial community monitoring as a promising approach to predict the ecological integrity of soil in the early stage of intensively managed tree plantations.

## Results

### Soil physicochemical properties, gaseous exchanges and abundance of bacteria

**Figure 1:**
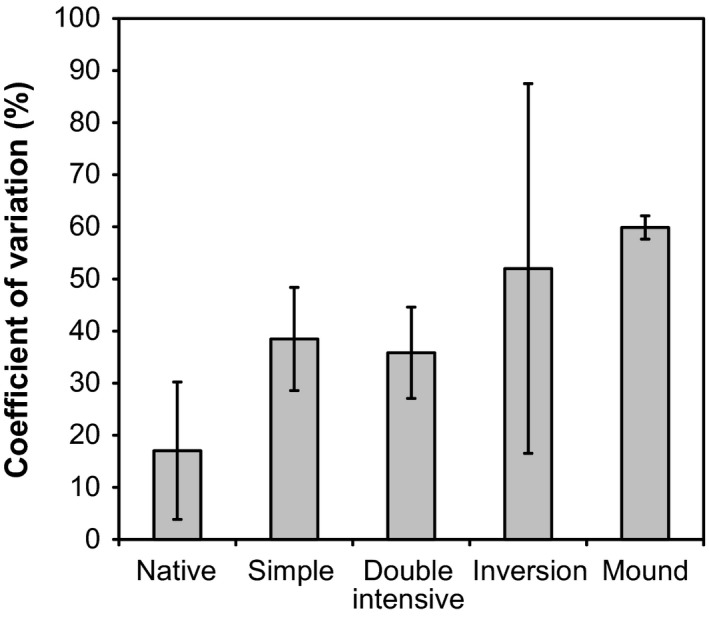
Coefficient of variation (CV) for gaseous exchanges. The bars represent the average and standard deviation of CV measured for H_2_, CO and CO_2_.

Conversion of unlogged natural forest to a hybrid larch plantation caused no significant change in measured soil physicochemical properties at the microsite level (Table 1). Soil carbon and nitrogen concentrations were significantly and positively correlated (Pearson, *P* < 0.0001) and C:N stoichiometry ranged from 18 to 32 among samples. All tested soil represented a net sink for H_2_ and CO and net source for CO_2_ (Table 1). Even though gaseous exchanges were not affected by MSP procedures, some treatments induced high variations between replicates. In general, the coefficient of variation (CV) of gas exchanges increased as a function of the intensification of the MSP techniques (Fig. 1). For instance, H_2_, CO and CO_2_ exchanges measured in soil samples collected in excavated mounds displayed CV ranging from 58% to 62% while the same variables measured in unlogged natural forest samples were characterized by CV of 7–32%. Trace gas turnover showed no significant relationship with soil carbon and nitrogen concentrations (Pearson, *P* > 0.05) but a negative correlation was observed between soil respiration and pH (Pearson, *P* = 0.03). H_2_ uptake rates increased as function of CO uptake (Pearson, *P* < 0.0001) and CO_2_ production (Pearson, *P* = 0.02) activities. CO_2_ production rates were not related to measured CO uptake activities (Pearson, *P* > 0.05). The abundance of bacteria was proportional to CO_2_ production rate in soil (Pearson, *P* = 0.01).

**Table 1:** Physicochemical properties, trace gas exchanges, bacterial 16S rRNA gene abundance and bacterial diversity in soil. Averages are represented with standard deviations in parentheses^a^

Sites	Carbon (%)	Sand (%)	Silt (%)	Clay (%)	Nitrogen (%)	pH	H_2_ oxidation (nmol g_(dw)_^−1^ h^−1^)	CO oxidation (nmol g_(dw)_^−1^ h^−1^)	CO_2_ production (μmol g_(dw)_^−1^ h^−1^)	16S rRNA gene (copies g_(dw)_^−1^)	Alpha diversity (Shannon)	Alpha diversity (ACE)
Unlogged natural	4.9 (1.1)	83 (3)	13 (3)	4 (1)	0.22 (0.04)	4.3 (0.1)	6.3 (0.4)	4.5 (0.6)	4.1 (1.3)	7.9 (1.3) ×10^9^	6.2 (0.08)	2950 (242)
Simple	5.4 (1.3)	86 (3)	13 (3)	1 (1)	0.27 (0.06)	4.6 (0.2)	5.8 (1.6)	4.5 (2.0)	3.1 (1.4)	4.9 (3.6) ×10^9^	6.3 (0.2)	2895 (565)
Double intensive	4.1 (1.2)	84 (2)	14 (2)	2 (0)	0.21 (0.06)	4.7 (0.2)	5.2 (1.4)	3.8 (1.7)	1.5 (0.5)	2.0 (2.6) ×10^9^	6.2 (0.3)	2617 (732)
Inversion	3.0 (1.4)	76 (2)	22 (2)	2 (0)	0.13 (0.04)	4.7 (0.2)	3.0 (2.3)	2.1 (1.4)	1.4 (0.2)	1.5 (1.4) ×10^9^	6.1 (0.5)	2268 (985)
Mound	4.3 (0.8)	85 (8)	14 (8)	1 (1)	0.17 (0.03)	4.6 (0.2)	6.6 (4.1)	4.8 (1.8)	1.1 (2.3)	1.8 (2.1) ×10^9^	6.0 (0.2)	2247 (877)

a No variable showed significant difference between the treatments (ANOVA, *P* > 0.05).

### Multifunctional soil classification

**Figure 2:**
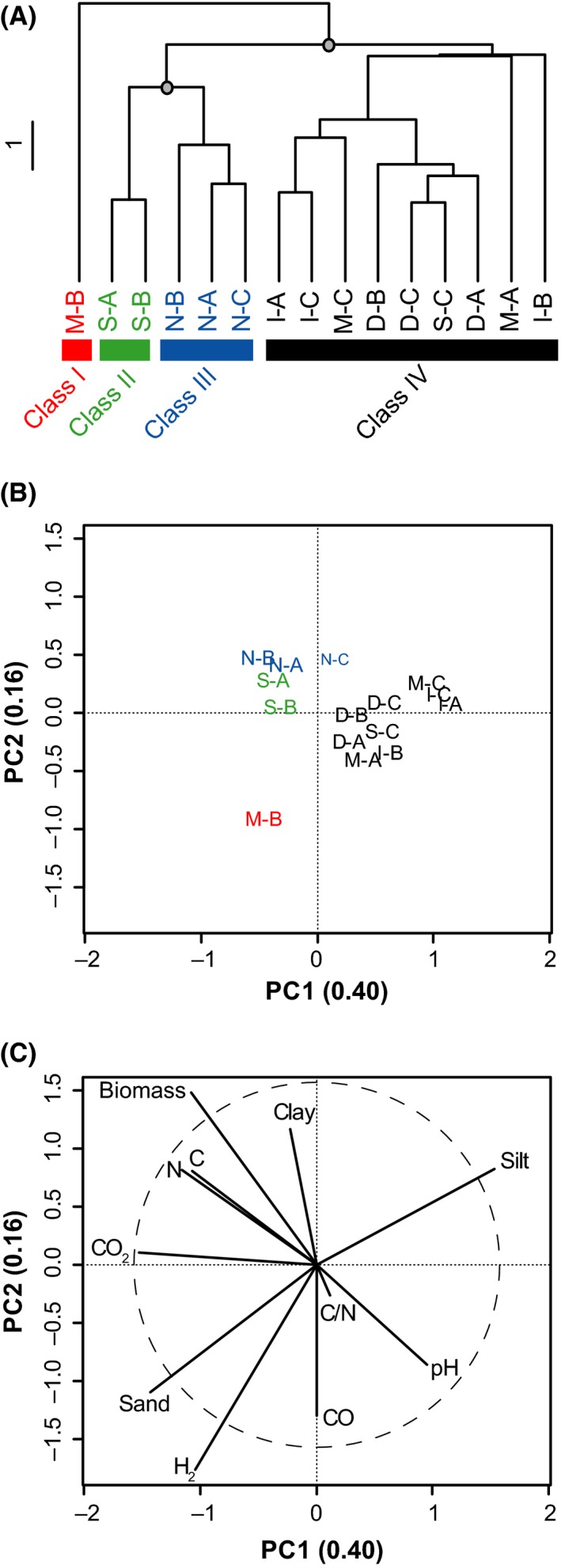
Multifunctional comparison of soil samples according to their physicochemical (C, N, C:N ratio, pH, texture), trace gas exchanges (H_2_, CO, CO_2_) and abundance of bacteria (16S rRNA gene abundance; labelled as ‘Biomass’ in the plot) profiles. (A) UPGMA agglomerative clustering of soil samples according to a Euclidean distance matrix calculated with standardized variables. The grey circles denote the nodes delineating the four multifunctional classes significantly discriminated by SIMPROF permutation procedure (*P* < 0.05). Colour labels show the assignation of the soil samples to their multifunctional class (red; class I, green; class II, blue; class II and black; class IV). The scale bar represents the Euclidean distance in the dendrogram. (B) Principal component analysis showing the distribution of sampling sites in a reduced space defined by soil physicochemical properties, gaseous exchanges and abundance of bacteria. The colours used to present soil samples correspond to the clusters identified in the UPGMA (A). (C) Variables defining the distribution of soil samples along the first and the second axis are represented along with the equilibrium circle of descriptors showing the contribution of variables to the formation of the reduced space. The detection limit of the qPCR assay was utilized to estimate the abundance bacteria in sample M‐A for which the low yield of the DNA extraction procedure precluded qPCR and bacterial 16S rRNA gene profiling (see the Material and Methods section for more details).

Soil physicochemical properties, gaseous exchanges and bacteria abundance variables were utilized to define a multifunctional soil classification. Under this classification approach, the distribution of each variable was considered to compute a distance matrix measuring the association between soil samples (Fig. 2A). Four different multifunctional classes were identified in the clustering analysis. The level of disturbance characterizing each class was defined on the basis of their Euclidean distance from the unlogged natural forest plots. Firstly, the soil sample constituting class I (M‐B) represented the MSP treatment that resulted in the most intense disturbance of baseline ecological functions. Secondly, soil samples included in class II (S‐A, S‐B) belong to the category of MSP treatments that caused slight deviations from baseline soil ecological functions. Soil samples belonging to class III (N‐B, N‐A, N‐C) correspond to baseline of soil ecological functions. Finally, class IV (S‐C, I‐A, I‐C, I‐B, M‐A, M‐C, D‐A, D‐B and D‐C) encompass soils for which the MSP treatments caused important alteration of baseline ecological functions at the microsite level. The unlogged natural forest was the sole condition for which replicated composite samples exhibited treatment‐specific, conserved multifunctional profile. Taken together, this classification model indicates that soil multifunctional profile observed in inversion and mound excavations plots were those showing the strongest deviation from unlogged natural forest at the microsite level. With the exception of one replicate (S‐C), simple trenching (S‐A and S‐B) was the treatment exerting the lowest incidence on soil multifunctional profile (Fig. 2A).

A principal component analysis was computed to represent the position of sampling sites in a reduced space defined by the measured variables. The ordination space defined by the first two components explained 56% of the variation observed (Fig. 2B). Five variables defined the reduced space represented by both axes (Fig. 2C). The gradient associated to CO_2_ production rate explained the distribution of plots along the first component, while abundance of bacteria and H_2_ uptake rate defined the distribution along the second axis. Soil CO_2_ respiration was the most preponderant variable responsible for the clustering of S‐A and S‐B trenching plots with unlogged natural forest samples and the clustering of S‐C with mounding, inverting and intensive trenching plots. Indeed, CO_2_ efflux was 3.9 μmol g_(dw)_^−1^ h^−1^ in S‐A and S‐B, while a value of 1.6 μmol g_(dw)_^−1^ h^−1^ was measured in S‐C. The sample M‐B displayed the highest H_2_ and CO uptake rates, explaining its position in the ordination space (Fig. 2B).

### Soil bacterial community taxonomic structure

**Figure 3:**
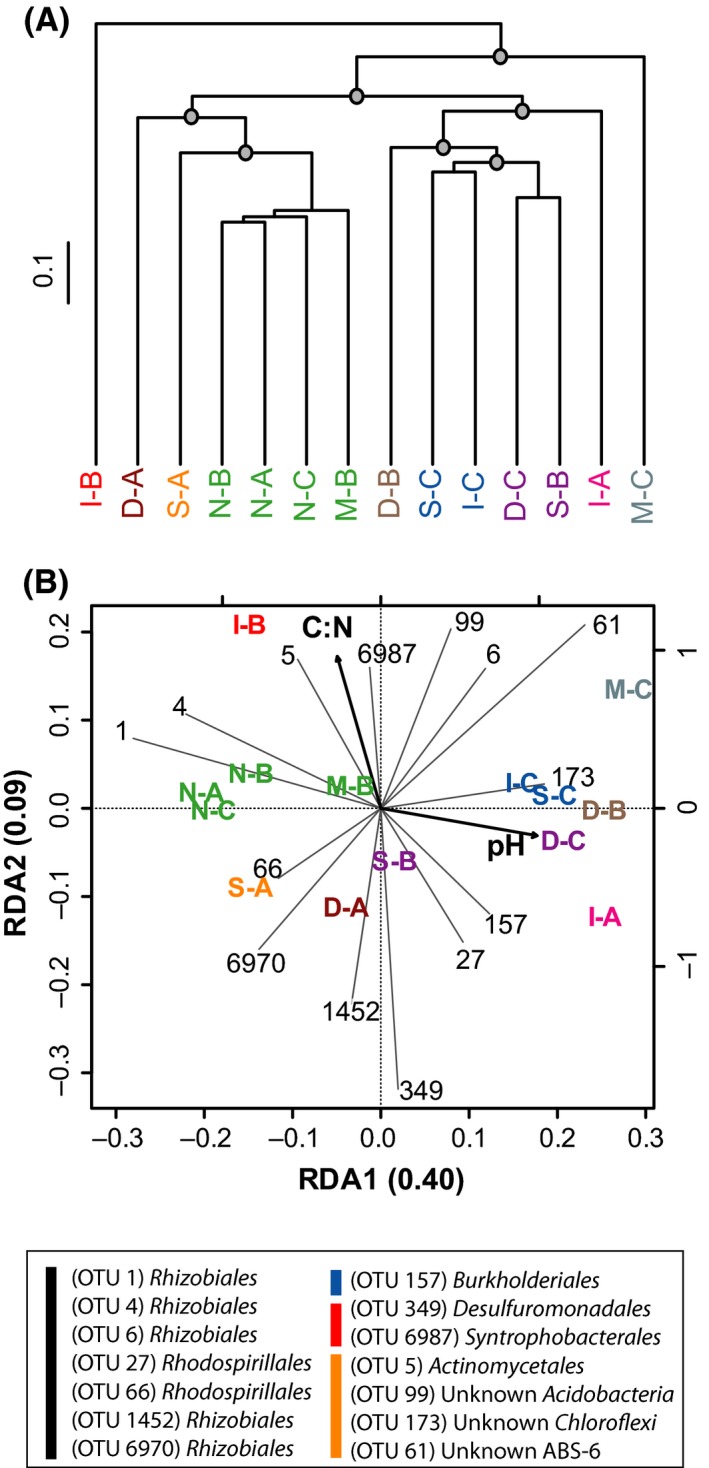
Comparison of soil samples according to their 16S rRNA gene profile. (A) UPGMA agglomerative clustering of soil samples derived from a matrix of Euclidean distance calculated after Hellinger transformation of OTU (97% identity threshold) absolute abundance. The grey circles denote the nodes delineating the four groups of samples significantly discriminated by SIMPROF permutation procedure (*P* < 0.05). The scale bar represents the Euclidean distance in the dendrogram. Colour labels show the assignation of the soil samples to their multifunctional class (red; class I, green; class II, blue; class II and black; class IV). (B) Parsimonious RDA triplot of Hellinger‐transformed OTU absolute frequency matrix explained by soil pH and C:N ratio. Only the 14 OTUs displaying extreme distribution in the reduced space are depicted for clarity. These OTUs are identified in the legend with colour bars discriminating *α‐Proteobacteria* (black), *β‐Proteobacteria* (blue), *δ‐Proteobacteria* (red) and other phyla (orange), as determined using the Greengene reference database V13_8_99 (McDonald *et al*., 2012). The colour labels used to present soil samples in the RDA triplot correspond to the clusters identified in the UPGMA (Fig. 4A). The sample M‐A is absent due the low yield of the DNA extraction procedure for this soil (see the Material and Methods section for more details).

Quality control, classification and equalization of the 16S rRNA gene sequence libraries yielded 5451 bacterial OTU (97% identity threshold). Overall, 50% of the sequences belonged to *Proteobacteria* mostly represented by *alpha*‐ (74%) and *delta‐Proteobacteria* (11%). The *Acidobacteria* and *Actinobacteria* were the two other phyla dominating the bacterial communities with 19% and 9% relative abundance respectively (Fig. S1). Neither the conversion of unlogged natural mixed forest to a hybrid larch monoculture nor MSP treatments caused significant alteration at the microsite level in bacterial OTU richness as evaluated by the Shannon diversity index and ACE estimator (Table 1). In general, beta diversity defined as the variability in OTU composition among replicated plots measured by multivariate dispersion showed more variations in the larch plantation than in unlogged natural mixed forest conservation areas (Fig. S2). Agglomerative clustering of the samples according to their microbial community profile showed that soil samples collected in different treatments could not be discriminated on the basis of OTU composition (Fig. 3A). Unlogged natural forest clustered together with M‐B, while all other clusters were composed either of unique plots or plots originating from different MSP procedures. A redundancy analysis (RDA) was performed to infer the relationship between 16S rRNA gene profiles and environmental variables (Fig. 3B). The most parsimonious model to explain variation in the distribution of 16S rRNA gene sequences included soil C:N stoichiometry and pH. The other variables being redundant to soil C:N and pH, their addition to the analysis increased the variance inflation factor unduly, and they were therefore ignored in the analysis. The first two canonical axes explained 49% of the total variance of bacterial OTU distribution. Significance of the RDA was confirmed with 1000 permutations of OTU data matrix (*P* = 0.001). Soil pH played an important role in the dispersion of the samples along the first axis, while C:N discriminated the samples along the second.

### Microbial molecular indicator for soil multifunctional classes

**Figure 4:**
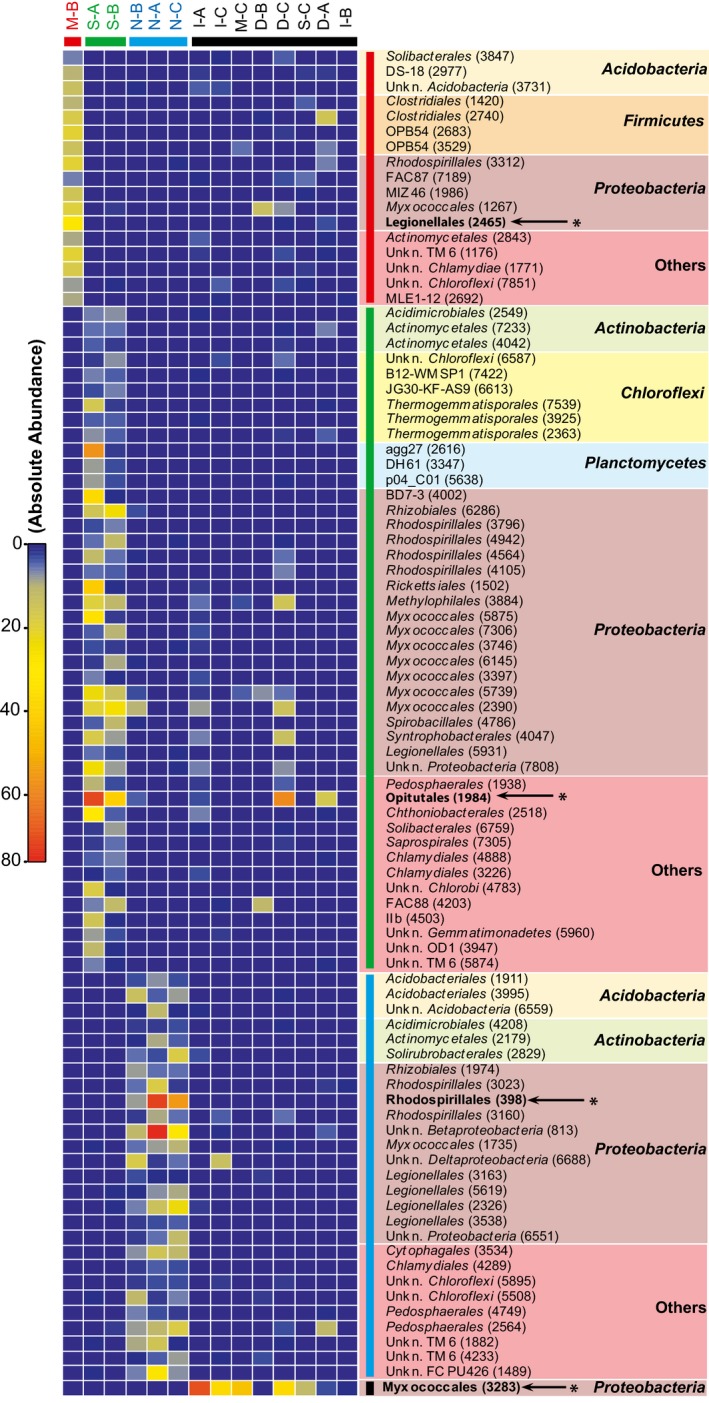
Identification of soil multifunctional molecular indicators. The heatmap shows the absolute abundance of OTUs detected in the soil samples categorized into the four multifunctional classes previously defined (Fig. 2A). Colour bars show the assignation of the soil samples to their multifunctional class (red; class I, green; class II, blue; class II and black; class IV). Taxonomic assignation of the OTU was done using the Greengene reference database V13_8_99 (McDonald *et al*., 2012). Representative indicators are highlighted with bold characters and are identified with an arrow and asterisk (← *).

The bacterial community profiles derived from the soil samples were classified within the four classes defined by the clustering of soil according to the multifunctional classification model (Fig. 2A). In total, 693 OTU were ubiquitously detected in all soil samples (Fig. S3). However, the search for indicator OTU unveiled coherence between the distribution of several members of the rare biosphere comprising less than 0.1% of the bacterial communities and soil multifunctional classes (Fig. 4). Indicator OTU encompassed a broad taxonomic diversity and *Proteobacteria* was the only phylum represented in the four multifunctional classes. In the case of multifunctional classes represented by more than one indicator, the OTU displaying the highest abundance was identified as representative indicator. Two indicators are of particular interest for this study. Firstly, the OTU 3283 (classified as a member of the order *Myxococcales*) was considered as a disturbance indicator because it was detected in soil samples for which the multifunctional classification (class IV) diverged from baseline ecological functions observed in unlogged natural mixed forest (Fig. 4). Furthermore, distribution of this candidate disturbance indicator was consistent with the environmental variables defining the multifunctional soil classification model. According to the unconstrained principal component analysis (PCA) ordination analysis, soil CO_2_ respiration was a preponderant variable discriminating soil samples classified in class IV from those belonging to class II and class III (Fig. 2). As expected, the abundance of 16S rRNA gene sequences belonging to OTU 3283 showed a negative correlation with CO_2_ soil respiration (Table 2). Secondly, the OTU 398 (classified as a member of the order *Rhodospirillales*) was considered as an indicator for baseline ecological conditions in soil because it was only detected in unlogged natural mixed forest conservation areas (Fig. 4). Distribution of this OTU was related to soil pH, bacteria abundance and soil clay content (Table 2). This result is in agreement with the contribution of these variables to the distribution of unlogged natural mixed forest soil samples in the PCA utilized to identify the factors defining the structure of the multifunctional classification model (Fig. 2B and C). In contrast to these two indicators, OTU for which the distribution was specific to multifunctional class I (OTU 2465; *Legionellales*) or class II (OTU 1984; *Opitutales*) did not show any significant correlation with the variables measured in this study (Table 2).

**Table 2:** Spearman correlation between the abundance of the representative indicator ribotype selected for the multifunctional classes identified in this study and soil biological, physical and chemical variables (see Table 1)

Variables	Selected multifunctional indicators			
	Class I OTU 2465 (*Legionellales*)	Class II OTU 1984 (*Opitutales*)	Class II OTU 398 (*Rhodospirillales*)	Class IV OTU 3283 (*Myxococcales*)
H_2_	0.34 (0.24)	0.03 (0.92)	0.25 (0.38)	**−0.67 (0.009)**
CO	0.05 (0.88)	−0.41 (0.15)	0.01 (0.98)	−0.53 (0.053)
CO_2_	0.14 (0.64)	0.16 (0.60)	0.49 (0.08)	**−0.77 (0.001)**
Bacteria abundance	−0.11 (0.70)	0.30 (0.29)	**0.68 (0.007)**	−0.46 (0.101)
C	−0.46 (0.10)	0.22 (0.44)	0.21 (0.47)	**−0.59 (0.026)**
N	−0.36 (0.21)	0.30 (0.29)	0.21 (0.47)	**−0.62 (0.017)**
C/N	−0.46 (0.10)	−0.45 (0.11)	0.40 (0.16)	−0.10 (0.74)
pH	0.13 (0.65)	−0.02 (0.95)	**−0.73 (0.003)**	**0.61 (0.020)**
Sand	0.43 (0.12)	0.44 (0.11)	0.06 (0.84)	**−0.72 (0.003)**
Clay	−0.07 (0.80)	−0.44 (0.12)	**0.81 (0.0005)**	−0.13 (0.65)
Silt	−0.39 (0.16)	−0.38 (0.19)	−0.21 (0.47)	**0.77 (0.001)**

Spearman rho correlation coefficients are presented with the significance levels (α‐value) in brackets. Bold characters represent significant correlation.

Two PCR assays were designed to challenge the indicator for baseline multifunctional conditions (OTU 398; class III) and the disturbance indicator (OTU 3283; class IV) identified through the indicator species statistical analysis (Table S1). Baseline indicator was detected in the three samples comprising class III (N‐A, N‐B, N‐C), but weak PCR amplification signal also was detected for sample M‐B (class I) and sample I‐A (class IV), where no reads belonging to OTU 398 where retrieved from the high‐throughput 16S rRNA gene sequencing analysis (Fig. S4A). On the other hand, disturbance indicator was detected in three out of the five samples from which reads assigned to OTU 3283 were detected in the sequencing analysis (Fig. S4B).

## Discussion

Monitoring of early growth of hybrid larch (*Larix* × *marschlinsii* Coaz) seedlings over two growing seasons following planting demonstrated that trenching (simple and double), mounding and inverting site preparation resulted in undistinguishable growth performance (Buitrago *et al*., 2015). This observation led to the conclusion that simple trenching represents the most economically attractive silvicultural practice for early growth of hybrid larch. Because soil microorganisms are at the core of key ecological functions such as nutrients transfer and biogeochemical cycles, we investigated the impact of MSP on the metabolic activity and composition of soil microbial communities. Our hypothesis that both the conversion of natural forest to a hybrid larch monoculture and the intensification of MSP treatments reduce the activity and the taxonomic diversity of soil microbiome in the early years of this conversion was not verified. Neither the microbial activities nor the diversity indices showed significant difference between the MSP techniques and natural unlogged forest. Beside the examination of environmental variables and the distribution of microorganisms in soil, the originality of our approach was the development of a multifunctional soil classification system along with molecular indicators to assess the potential ecological risk of MSP techniques on ecosystem functions. The integration of biogeochemical and microbiological variables – instead of considering variables individually – was shown to be essential to determine the response of soil bacterial community to silvicultural practices.

An important question in conservation biology is the number of species that must be protected to ensure ecosystem functioning. A milestone in the field of biodiversity and ecosystem functioning was the observation that the minimal number of species required to support multiple functions is much higher than estimates derived from the analysis of individual processes (Hector and Bagchi, 2007; Gamfeldt *et al*., 2008). As ecosystems are conserved for their multiple natural services, these studies highlighted the need for integrated approaches to set reasonable conservation objectives (Balvanera *et al*., 2014). In this study, we selected multiple gas turnover as a subset of ecosystem functions. H_2_ and CO soil uptake activities are catalysed by different guilds of bacterial species that contribute to mitigate the global emissions of these climate‐relevant trace gases in the atmosphere. High‐affinity, H_2_‐oxidizing bacteria are mostly represented by specialized *Actinobacteria* and some representatives of the *Proteobacteria*,* Chloroflexi* and *Acidobacteria* (Constant *et al*., 2011; Meredith *et al*., 2013). These bacteria are responsible for 80% of the global sink of atmospheric H_2_ (Constant *et al*., 2009; Ehhalt and Rohrer, 2009). Soil survey showed the importance of carbon and nitrogen contents to explain the activity of this functional group in soil (Gödde *et al*., 2000; Khdhiri *et al*., 2015). Carboxydovore bacteria, encompassing the *Actinobacteria*,* Proteobacteria* and *Chloroflexi*, are responsible for the soil uptake of atmospheric CO (King and Weber, 2007; King and King, 2014; Quiza *et al*., 2014). Even though no systematic investigation about the impact of soil nutrients on CO uptake rate has been reported, organic matter‐rich soils displayed higher potential CO uptake activity than desert soils along vegetation transects (Weber and King, 2010). The activity of carboxydovore bacteria contributes to 15% of the global sink of atmospheric CO, while scavenging CO produced by biological and abiotic reactions in soil. In contrast to H_2_ and CO, soil CO_2_ respiration is supported by a broad diversity of bacteria and fungi thriving in soil using a heterotrophic growth metabolism. In addition to the availability of soil organic matter, factors influencing soil respiration rate are complex, including soil nutrient stoichiometry (Drake *et al*., 2013). Including H_2_, CO and CO_2_ exchanges in multifunctional classification model was, therefore, a relevant approach to consider important ecosystem services that are provided by taxonomically diverse microorganisms. Addition of further environmental variables such as pH, C, N, abundance of bacteria and soil texture defining soil biogeochemical structure provided a supplementary dimension to the multifunctional classification system. Consideration of other environmental variables such as organic matter and nutrient turnover in the future is not precluded as this could refine the soil multifunctional classification system to satisfy other ecosystem functions prioritized in sustainable forest management.

In contrast to the comparative analysis of individual environmental parameters that unveiled no significant impact of MSP due to the variance of the observations, the multifunctional classification approach led to the identification of MSP treatment plots for which ecological functions differed from baseline conditions as measured in unlogged natural forest nearby. According to this classification, double trenching, mounding and inverting site preparation were the less sustainable MSP techniques at the microsite level. The low environmental performance of mounding and inversion plots at this scale was also supported by the higher coefficients of variation in the gaseous exchanges measured in these soils than those observed in trenching and unlogged natural mixed forest (Fig. 1). This finding, indicating that three distinct ecological services were sensitive to mounding and inverting, is probably due to the heterogeneity caused by the heavy machinery used for site preparation. The procedure involved the transfer of excavated soil and was prone to large variations due to the presence of rocks, resulting in variable volume and depth of the excavations in addition to potential variation in the horizontal and lateral distribution of displaced organic and mineral soil horizons and ground microtopography. Our results are relevant at the microsite level, *i.e*. local environmental conditions directly influencing planted seedling physiology and growth. Although mounding and inverting intensively disturb the soil profile, thus having significant local impacts on soil functions, trenching furrows affect a higher proportion of the planted site. Determining which treatment is best in terms of overall ecological impacts will require further work, including scaling up and interpreting these impacts at the stand level, taking into account the proportion of disturbed soil in each treatment.

Soil microbial diversity was neither related to MSP treatment nor multifunctional classes identified in this study. Soil pH, rather than MSP treatments, was the most important parameter to explain the composition of soil microbial communities (Fig. 3). Indeed, variation in pH was shown as a preponderant variable explaining the composition of soil microbiome and any silvicultural treatment resulting in marked pH alteration are expected to influence the distribution of dominant lineages (Lauber *et al*., 2009). The most important aspect of soil microbial community analysis was the clustering of the 16S rRNA gene profiles according to the multifunctional soil classification model, resulting in the identification of indicator OTU. Those indicators represented members of the rare biosphere in soil, for which the incidence of environmental variables on their distribution could not be observed using ordination techniques parameterized with the whole microbial community profiles (Fig. 3). Two relevant indicators were identified. The OTU 3283 (*Myxococcales*) detected in soil samples that were divergent from the baseline conditions observed in unlogged natural mixed forests could be used as a diagnostic tool to assess the impact of MSP on ecosystem services. On the other hand, the OTU 398 (*Rhodospirillales*) was associated to baseline soil multifunctional attributes. Bioindicators of soil quality involving exoenzyme activity measurements (Staddon *et al*., 1999) and other approaches involving the monitoring of arthropods (Pearce and Venier, 2006) and small mammals (Pearce and Venier, 2005) as bioindicators of sustainable forest management have already been developed. In contrast to these metrics requiring relatively laborious monitoring efforts, molecular diagnostic tools, specific to selected indicator OTU, are fast and easily integrated with abiotic and biological factors in the environment. In the context of the hybrid larch plantation investigated here, monitoring of the identified indicators, especially the indicator for baseline multifunctional soil attributes, could be used for environmental certification and ecological risk assessment after adequate scaling up addressing key requirements of ecological indicators.

The molecular indicators identified in this study are relevant at the stand level, with the abiotic and biotic conditions that prevailed in soil at the time of sampling. The experimental plan was not designed to address the consistency of molecular indicators over time, space and sites. Further investigations are needed to challenge the indicators in other hybrid larch as well as other fast‐growing tree plantations and assess their specificity (Bartell, 2006). Longer term studies are also needed to assess whether spatial and temporal variations of soil biogeochemical processes in intensive silviculture can be related to alterations of molecular indicator distribution profiles. Indeed, it is expected that indicator OTU identified in this study are not universal due to the impact of abiotic and biological gradients shaping soil microbial communities in soil. Nevertheless, our data show that the distribution of soil microbial communities is not random, with the distribution of some members restricted to specific multifunctional soil classes, at the stand level. Combination of soil bacterial, fungal and archaeal community taxonomic and functional profiles through metagenomic surveys should be considered in future attempts to develop and validate molecular indicators for sustainable forest management. Finally, PCR diagnostic assays targeting the baseline and stress indicators clearly demonstrated that a composite of indicator OTU rather than a single indicator would be needed for predicting soil multifunctional classes (Fig. S4). This could be achieved through the adaptation of the indicator species modelling approach used to select the minimal subset of species necessary to predict species richness of invertebrate and vertebrate assemblages to microbial ecology (Thomson *et al*., 2007; Azeria *et al*., 2009).

In conclusion, we showed that MSP treatments influence the overall signature of soil biogeochemical structure at the microsite level, suggesting that mounding and inverting site preparation could pose higher potential ecological risk for ecological functions in soil than trenching at the local scale. Further work is needed to scale up and interpret these results at the landscape level and over longer period of time, taking into account the proportion of disturbed soil in each treatment and the overall productivity of each treatment. In effect, the lower ecological risk potential of a less productive treatment could be cancelled by the need of converting a larger area into fast‐growing plantations to make up for the lesser productivity. Chronosequences, spatial variability, and different tree plantation types will need further attention in future investigations to elucidate the short term impact of MSP on soil biogeochemical structure and challenge the molecular indicators identified in this study. Because of functional redundancy in microorganisms, these studies would be essential to challenge and define limitations of the indicators. Nevertheless, this work demonstrates the relevance of applied ecology to evaluate the sustainability of silvicultural practices.

## Experimental Procedures

### Sampling site

**Figure 5:**
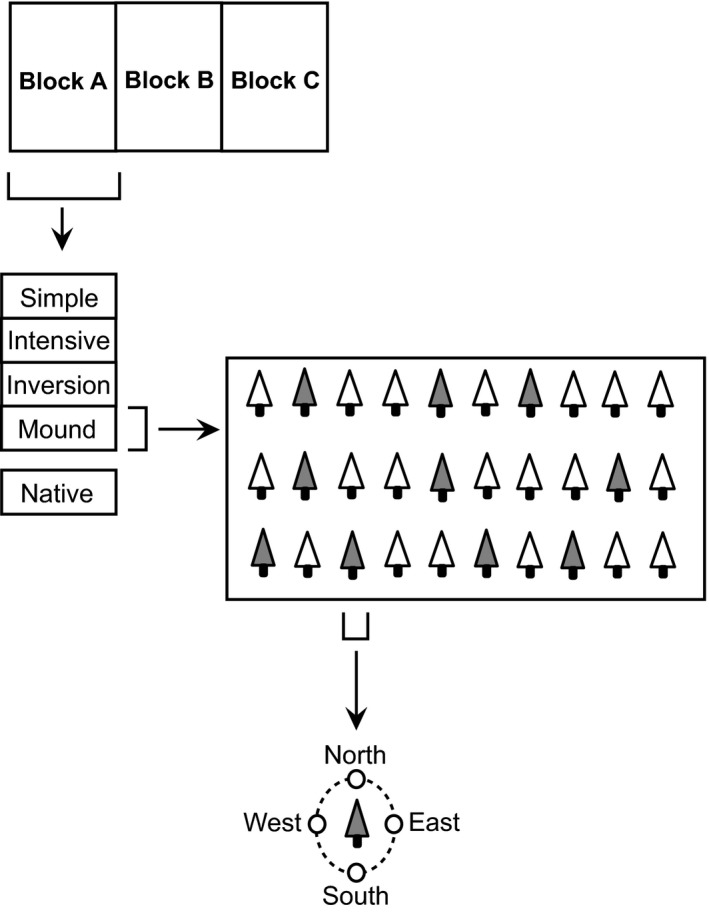
Schematic representation of the sampling design. Five treatment plots were replicated in three blocks. Soil samples were collected at 10 locations from each plots (illustrated with grey trees). Soil was collected in proximity of the stem, at one of the four cardinal points. Replicated soil samples were pooled to obtain one composite sample per replicated plot. The (native) treatment consisted of non‐harvested, non‐planted natural mixed forest areas located within the experimental blocks.

The study site was located near La Tuque (Québec, Canada; 47° 37′ 19″ N, 72° 49′ 55″ W), about 250 km north of Montréal. The experimental area, dominated by balsam fir (*Abies balsamea* (L.) Mill.), paper birch (*Betula papyrifera* Marsh.), yellow birch (*B. alleghaniensis* Britt.), red maple (*Acer rubrum* L.) and black spruce (*Picea mariana* (Mill.) BSP) was harvested in 2009 (clear‐cut with 5% retention) prior to the installation of an experiment to investigate the impact of different MSP techniques on the growth performance of hybrid larch planted in April 2010 (Buitrago *et al*., 2015). The experimental design consisted of a complete block design comprising three replicated blocks (Fig. 5). Briefly, each block was separated into four plots randomly assigned to different MSP treatments encompassing trenching and mounding. Trenching was performed with rotary discs mixing mineral and organic soil horizons. This treatment was applied either as a simple (simple plots; composite samples S‐A, S‐B, S‐C) or double trenching (double intensive plots; composite samples D‐A, D‐B, D‐C) corresponding to an increasing gradient of soil mixing (Buitrago *et al*., 2015). An excavator was used for the mounding treatments consisting in inverting the soil horizons to place mineral soil on the top and the organic layer at the bottom. Excavated soil was either placed back to the original ditch (inversion plots; composite samples I‐A, I‐B, I‐C) or on soil surface next to the ditch generated by the excavation (mound plots; composite samples M‐A, M‐B, M‐C). Seedlings were planted in the hinge position of the trenching furrows or near the centre of excavated mounds. Finally, plots consisting of non‐harvested, non‐planted natural mixed forest areas (retention areas located within the three experimental blocks, and consisting in approximately 500–600 m^2^ plots) were used as reference in this study (unlogged natural plots; composite samples N‐A, N‐B, N‐C). These unlogged forest soils were selected as reference plots based on third‐party forest certification criteria (*e.g*. Forest Stewardship Council), where reference forests in the vicinity of forest management units are used to measure the impact of management plans on ecosystem integrity.

The experimental area was visited in July 2014 for soil sample collection. In total, 10 replicated soil samples were collected in each plot (3 blocks × 5 plots × 10 replicates = 150 samples in total). Individual trees for which information regarding early growth parameters was available (Buitrago *et al*., 2015) were first randomly selected and the A‐horizon (0–10 cm) was collected within a 15 cm radius of the stem at one of the four cardinal points also determined randomly (Fig. 5). Each replicate sample consisted of ~200 g soil stored on ice in Whirl‐Pak^®^ bags. Composite samples (3 blocks × 5 plots = 15 composites) were prepared on the site less than 6 h after collection. Approximately, 100 g soil from each replicate were thoroughly mixed in a plastic bucket and transferred to plastic bags. All the samples were then immediately stored at 4°C. The 15 composite samples were processed within 3 weeks for DNA extraction, nutrient analyses and gas exchanges measurements. Soil texture was determined using the hydrometer method, and particle size distribution assigned soil samples to the loamy sand textural class (Elghamry and Elashkar, 1962). Because soil samples were collected in a 15‐cm radius of the stem, the results reported in this study are representative of a microscale of the whole experimental larch plantation landscape area.

### Physicochemical analyses

Composite soil samples were dried at 20°C for 48 h, homogenized (2 mm sieve) and pulverized with mortar and pestle. Soil pH was analysed in suspensions using a 1:2.5 (w/v) soil‐to‐water ratio (MP220 pH‐meter; Mettler Toledo, Mississauga, ON, Canada). Total soil carbon and nitrogen contents were determined using an elemental combustion system (ECS 4010; Costech Analytical Technologies Inc., Valencia, CA, USA). Analyses were performed with 18 to 123 mg pulverized soil samples and certified atropine standard (C_17_H_23_NO_3_; Costech Analytical Technologies Inc.) containing 70.86% total carbon and 4.84% total nitrogen was used for calibration. Relative errors were lower than 2.8% for total carbon and 1.1% for total nitrogen analyses as observed with repeated analyses of atropine standard.

### Gaseous exchanges

Composite samples were dried at 20°C for 48 h and homogenized (2 mm sieve) and soil water content was adjusted to 20% water holding capacity. A defined amount of composite soil sample (20 g) was transferred in a 500 ml (nominal volume) Gibco^®^ glass bottle (Wheaton Industries Inc., Millville, NJ, USA) fitted with foam plugs to allow gaseous exchanges between soil and atmosphere, while avoiding microcosm contamination with airborne particles. Soil microcosms were then transferred to an environmental chamber (MLR‐350^®^; Sanyo, Osaka, Japan) and incubated 3 days in the dark, at 25°C and 50% relative air moisture. H_2_, CO and CO_2_ soil‐to‐air exchanges were measured using gas chromatography assays. Briefly, soil microcosm foam plugs were replaced with gastight caps equipped with butyl septa. A defined volume of two air mixtures containing either 469 ± 9 ppm H_2_ (GTS‐Welco, Minersville, PA, USA) or 508 ± 10 ppm CO (GTS‐Welco) was injected to the static headspace of the microcosms, resulting in H_2_ and CO levels of 2.5–3 ppmv. Headspace samples (10 ml) were collected with a Pressure Lok^®^ gastight glass syringe (VICI^®^ Precision Sampling Inc., Baton Rouge, LA, USA) and injected through the injection port of a gas chromatograph equipped with a reduction gas detector (ta3000R; Ametek Process Instruments^®^, Newark, DE, USA). The first‐order oxidation rates were calculated by integrating the H_2_ and CO mole fraction time series measured over a 1‐h period, using at least five concentration points for data integration. Soil CO_2_ respiration was measured in the soil microcosms fitted with gastight caps and flushed with ambient laboratory air. Linear CO_2_ mole fraction time series was measured over a 72‐h period, with four concentration points for data integration using a gas chromatograph equipped with a methanizer and a flame ionization detector (7890B GC System; Agilent Technologies, Mississauga, ON, Canada). Sealed microcosms were incubated in the environmental chamber during the measurements. The reproducibility of gas analyses was assessed before each set of experiments by repeated analysis of three certified standard gas mixtures: (i) 2.13 ± 0.11 ppmv H_2_ balance air (GTS‐Welco), (ii) 2.05 ± 0.10 ppmv CO balance air (GTS‐Welco) and (iii) 610 ± 12 ppmv CO_2_, 5 ± 0.1 ppmv CH_4_, 1 ± 0.02 ppmv N_2_O balance nitrogen (Agilent Technologies), and standard deviations were < 5%. No significant gaseous exchanges were observed for blank measurements of empty microcosms. Because of the occurrence of simultaneous production and consumption of trace gases in nature and their dependence on temperature and moisture, gaseous exchanges presented in this study must be considered as potential activities. Gaseous exchanges were expressed in mol of gas h^−1^ g^−1^ of soil on a dry‐weight basis; with soil water content measured using standard gravimetric method.

### Abundance of bacteria

The abundance of bacteria was estimated by quantification of 16S rRNA gene of bacteria in soil by a universal bacterial qPCR assay (Fierer *et al*., 2005). Genomic DNA was extracted from 0.5 to 3.0 g_(dw)_ composite soil samples using a combination of chemical and mechanical cell lysis procedure (Constant *et al*., 2008). DNA was precipitated with 2 volumes 96% ethanol and polyvinylpolypyrrolidone spin column was used for final purification (Berthelet *et al*., 1996). Purified DNA extracts from two technical replicates were pooled (200 μl in total) and kept frozen at −20°C before qPCR. The reactions were performed using 5 μl of diluted genomic DNA (1:100 and 1:500) and no quantification bias due to Taq polymerase inhibitors was observed. The qPCR assay was based on a standard curve prepared by using triplicate 10‐fold dilutions of PCR‐amplified standard DNA. Genomic DNA of *Burkholderia xenovorans* LB400 served as template for 16S rRNA gene standard DNA. PCR products were purified (E.Z.N.A. Cycle Pure Kit, Omega Bio‐Tek^®^, Norcross, GA, USA) and quantified using the Quantifluor^™^ dsDNA System (Promega, Madison, WI, USA) according to the instructions of the manufacturers. Standard curves encompassing 10^2^ to 10^8^ copies μl^−1^ of standard DNA were prepared and displayed linear relationship between the signal and the logarithm copy number with reaction efficiencies of 0.99 (*r* ^2^ = 0.99). The Perfecta SYBR Green Fast Mix (Quanta Biosciences^®^, Gaithersburg, MD, USA) was used for the qPCR performed in a Rotor‐Gene 6000 qPCR cycler (Corbett Life Science^®^, Concorde, NSW, Australia). For unknown reasons, the extraction procedure was not successful for the sample M‐A. Neither the utilization of FastDNA SPIN kit for Soil^®^ (MP Biomedicals, Solon, OH, USA), modifications of the extraction buffer, nor increased amount of soil in the lysis procedure improved the yield of the extraction for sample M‐A. This sample thus was not considered for qPCR and bacterial 16S rRNA gene ribotyping analyses.

### Bacterial 16S rRNA gene sequencing

PCR amplification of the V6‐V8 regions of 16S rRNA, libraries preparation and Illumina MiSeq 250 bp paired‐ends sequencing reactions were performed by the technical staff of *Centre d'Innovation Génome Québec et Université McGill*, resulting in 6 274 978 raw sequences. Paired‐end reads were merged using the software Flash (Magoč and Salzberg, 2011) with minimum and maximum overlap length between the two reads of 20 and 250 bases respectively. The maximum proportion of mismatched base pairs tolerated in the overlap region was 30%. The 6 073 727 merged reads were processed using the software UPARSE (Edgar, 2013). Briefly, sequences were truncated to a uniform length of 420, representing the length of more than 97.5% of the sequences in the database. Reads with a low‐quality score were removed using 2.0 as the maximum expected error value. The remaining 4 884 667 high‐quality reads were de‐replicated, sorted by size and singletons were removed. The remaining unique reads were clustered into 8248 operational taxonomic units (OTU) with the UPARSE‐OTU greedy clustering method using a 97% identity threshold. The UPARSE‐REF algorithm detected and removed 98 006 chimeric sequences during clustering procedure. Furthermore, 191 chimeric OTU were removed with the software UCHIME run against ChimeraSlayer ‘gold’ reference database (Edgar *et al*., 2011). The final database contained 4 228 736 sequences. Libraries were normalized to the sequencing effort of the smallest 16S rRNA gene library (166 040 sequences) to avoid biases in comparative analyses introduced by sampling depth. The software QIIME version 1.8.0 (Caporaso *et al*., 2010) was used to perform equalization of the libraries and to eliminate OTU comprising less than eight representative sequences, corresponding to a threshold of 0.005% of the total number of reads per library. QIIME was also used to pick and align one representative sequences for each OTU to assign a taxonomic classification using the Greengene reference database V13_8_99 (McDonald *et al*., 2012). The resulting OTU table comprising the abundance and taxonomic affiliation (phylum, class, order and family levels) of the OTUs in the samples was utilized to compute alpha diversity (*i.e*. species richness with Ace and Shannon indices) and the multivariate dispersion of the OTU as a measure of beta diversity (Anderson *et al*., 2006) with the packages ‘fossil’ and ‘vegan’ implemented in the software R (Oksanen *et al*., 2012; Vavrek, 2012). Raw sequences were deposited to the Sequence Read Archive of the National Center for Biotechnology Information under the Bioproject PRJNA280109.

### Statistical analyses

Statistical analyses were performed using the software R (R Core Development Team, 2008). The impact of MSP treatments on soil biogeochemical properties was tested using one‐way analysis of variance and *post hoc* Bonferroni‐corrected *t*‐test. Shapiro–Wilk normality test was applied to assess normal distribution of data before the ANOVA. Distribution of soil pH, carbon content and nitrogen content followed a normal distribution, while the distribution of other variables was normalized by logarithmic (H_2_, CO, CO_2_ exchanges) or square root (abundance of bacteria) transformations. Normalized variables also were used for Pearson correlation analysis. Cluster analysis was computed using the function ‘hclust’ in the package ‘stats’ (R Core Development Team, 2014) to explore sampling site similarities defined by variations in gas exchange (H_2_, CO and CO_2_), pH, abundance of bacteria as well as total carbon and total nitrogen contents measured in soil. The environmental variables were standardized before the analysis by subtracting individual values by the average and dividing them by the standard deviation. A Euclidean distance matrix was used to generate a UPGMA agglomerative clustering of the samples according to their biogeochemical profile. The identification of statistically different soil multifunctional classes was done by performing 999 permutations of the environmental variables data set separately across the samples and comparing the observed similarity score of each cluster against the expected values under the null hypothesis using the similarity profile tool (SIMPROF) implemented in the package ‘clustsig’ (Clarke *et al*., 2008). PCA was used to explore sampling sites partitioning in a reduced space defined by environmental gradients. Meaningful ordination axes whose eigenvalues were larger than the average of all eigenvalues were selected for biological interpretation. Equilibrium circle of descriptor with the radius $\sqrt{d/p}$ (where *d* is the number of dimensions of the reduced space: 2 and *p* is the total space: 10) was plotted to identify variables significantly contributing to the axes defining the position of sampling sites. Discrimination of the samples according to their ribotyping profile was performed after Hellinger transformation of the OTU table to avoid unduly relationships between explanatory variables and 16S rRNA gene composition supported by the high weight of rare species (Legendre and Gallagher, 2001). UPGMA clustering analysis was conducted to compare the samples according to their ribotyping profile. RDA was computed using standardized environmental variables with the package ‘vegan’ (Oksanen *et al*., 2012), according to the comprehensive procedure described by Borcard and colleagues (Borcard *et al*., 2011). The most parsimonious constrained model to explain bacterial ribotyping profile was obtained by forward selection of the environmental variables (Blanchet *et al*., 2008) and permutation tests (*n* = 1000) were performed to assess the significance of the RDA. Indicator OTU characterizing UPGMA agglomerative clustering of the samples according to their multifunctional classification were identified using the indicator species analysis procedure implemented in the package ‘indicspecies’ (Dufrêne and Legendre, 1997). Minimal significance level (alpha) of the indicator OTU was 0.05, tested against 999 random permutations of samples among the biogeochemical clusters.

## Conflict of Interest

All the authors have no conflict of interest to declare.

## Supporting information


**Fig. S1.** Distribution of the ribotypes classified at the phylum taxonomic level.
**Fig. S2.** Beta diversity of the ribotypes (OTU defined at 97% identity threshold) as assessed using multivariate dispersion measure (Anderson *et al*., 2006).
**Fig. S3.** Relative abundance of ubiquitous ribotypes detected in all soil samples (OTU were classified at the phylum level).
**Fig. S4.** PCR detection of (a) OTU 398 and (b) OTU 3283 identified as potential indicator for soil samples characterized by baseline multifunctional attributes and soil samples that are divergent from baseline attributes respectively.
**Table S1.** Oligonucleotides and PCR conditions utilized to detect bioindicator for baseline soil multifunctional attributes and soil samples that are divergent from the baseline multifunctional attributes.
