# Supplementary material for: Towards the development of multifunctional molecular indicators combining soil biogeochemical and microbiological variables to predict the ecological integrity of silvicultural practices
Source: Microb Biotechnol. 2016 Feb 8;9(3):316–29. doi: 10.1111/1751-7915.12348 (PMC4835570; doi:10.1111/1751-7915.12348)
Supplement: Supplementary file 1 — Fig. S1. Distribution of the ribotypes classified at the phylum taxonomic level. Fig. S2. Beta diversity of the ribotypes (OTU defined at 97% identity threshold) as assessed using multivariate dispersion measure (Anderson et al., 2006). Fig. S3. Relative abundance of ubiquitous ribotypes detected in all soil samples (OTU were classified at the phylum level). Fig. S4. PCR detection of (a) OTU 398 and (b) OTU 3283 identified as potential indicator for soil samples characterized by baseline multifunctional attributes and soil samples that are divergent from baseline attributes respectively. Table S1. Oligonucleotides and PCR conditions utilized to detect bioindicator for baseline soil multifunctional attributes and soil samples that are divergent from the baseline multifunctional attributes. [file MBT2-9-316-s001.docx]

**Supplementary materials**

The next sections present the relative abundance of the ribotypes classified at the phylum level (Figure S1A). Distribution of ribotypes classified as α-, β-, δ- and γ-*Proteobacteria* is also shown (Figure S1B). Multivariate dispersion of the ribotyping profile obtained for each MSP method and native mixed forest is presented to assess beta diversity (Figure S2). Relative abundance of the ubiquitous ribotypes detected in all soil samples, classified at the phylum level (Figure S3). PCR detection of potential indicators for soil samples characterized by baseline multifunctional attributes and soil samples that are divergent from baseline attributes was performed to challenge the conclusion of the indicator species statistical analysis (Figure S4). Oligonucleotides and PCR conditions utilized to detect the two indicators are presented in table S1.

**Figure S1.** Distribution of the ribotypes classified at the phylum taxonomic level.


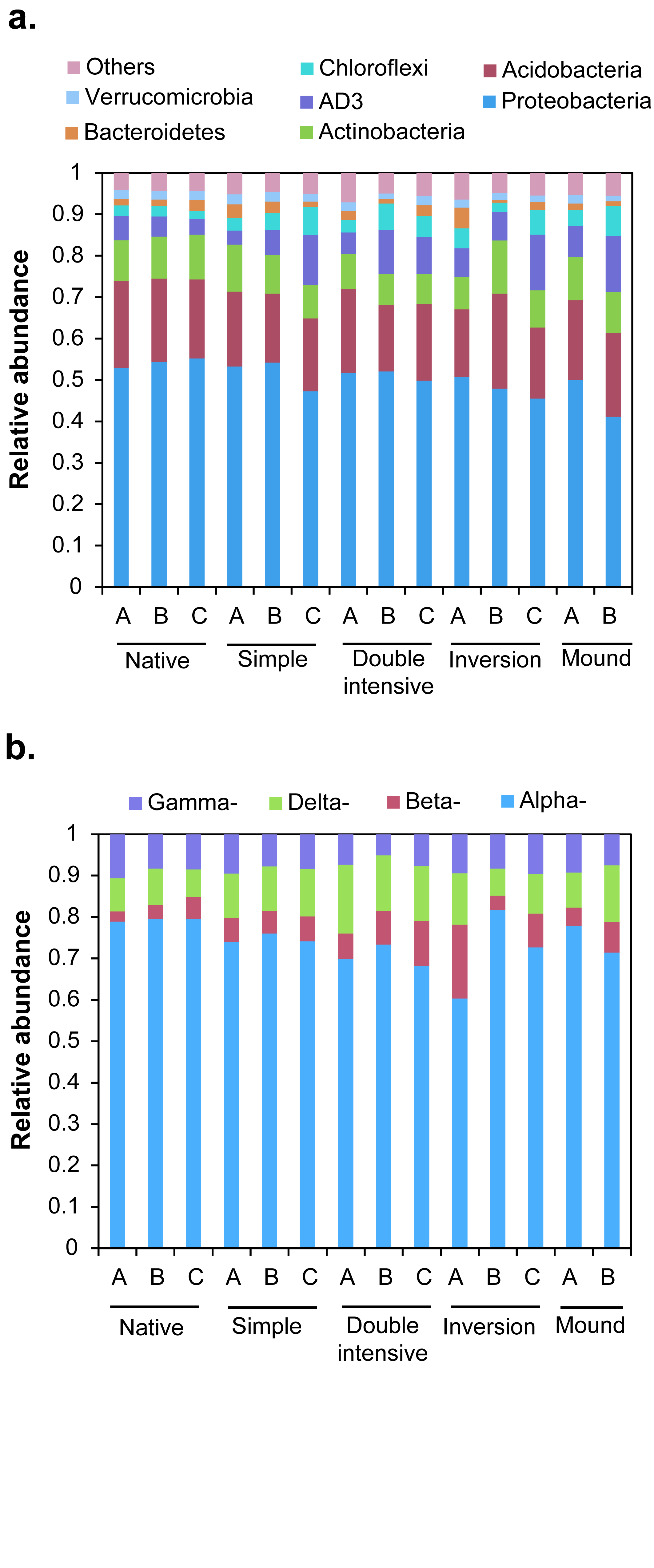


**Figure S2.** Beta diversity of the ribotypes (OTU defined at 97% identity threshold) as assessed using multivariate dispersion measure ([Anderson et al., 2006](#_ENREF_1)). A test of the null hypothesis of no difference in the multivariate distribution of the four groups using deviation from centroid and 999 permutations of the least-square residuals was computed using the package Vegan implemented in R ([Oksanen et al., 2012](#_ENREF_2)). Because the null hypothesis was accepted (F = 1.34, α = 0.35), variability in species composition among the sample units representing each MSP methods and native mixed forest does not differs significantly. The boxplot shows calculated multivariate distance, where the lower and upper values are represented with the whiskers and the median is represented with the black bar for each group. The absence of whiskers for mounding was due to the utilization of two instead of three replicates (the sample M-A was absent due the low yield of the DNA extraction procedure for this soil - see the material and methods section for more details).


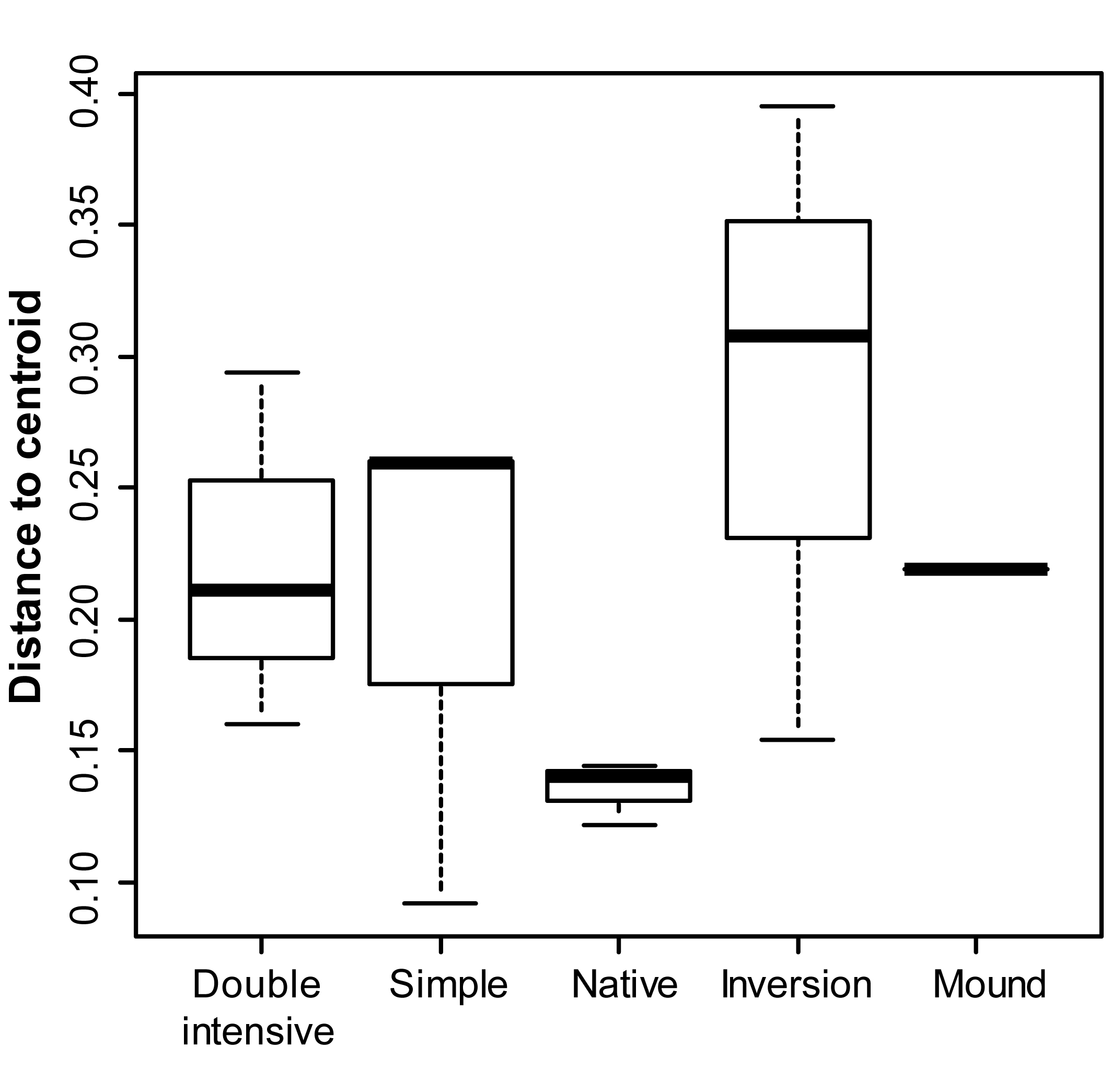


**Figure S3.** Relative abundance of ubiquitous ribotypes detected in all soil samples (OTU were classified at the phylum level).


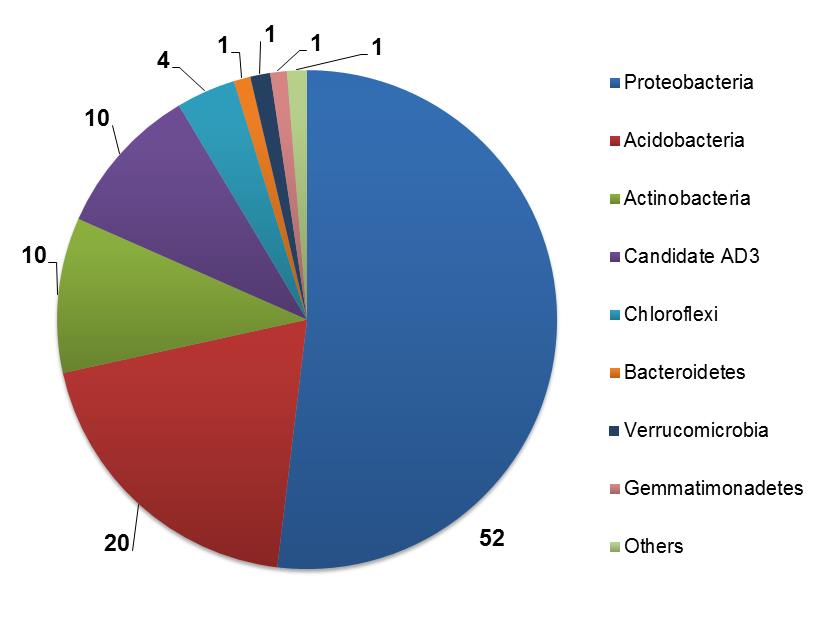


**Figure S4.** PCR detection of (a) OTU 398 and (b) OTU 3283 identified as potential indicator for soil samples characterized by baseline multifunctional attributes and soil samples that are divergent from baseline attributes, respectively. The red asterisks (*) denote false positive and false negative PCR signal when compared to 16S rRNA high throughput sequencing (see the heatmap reported in the figure 5 of the article).


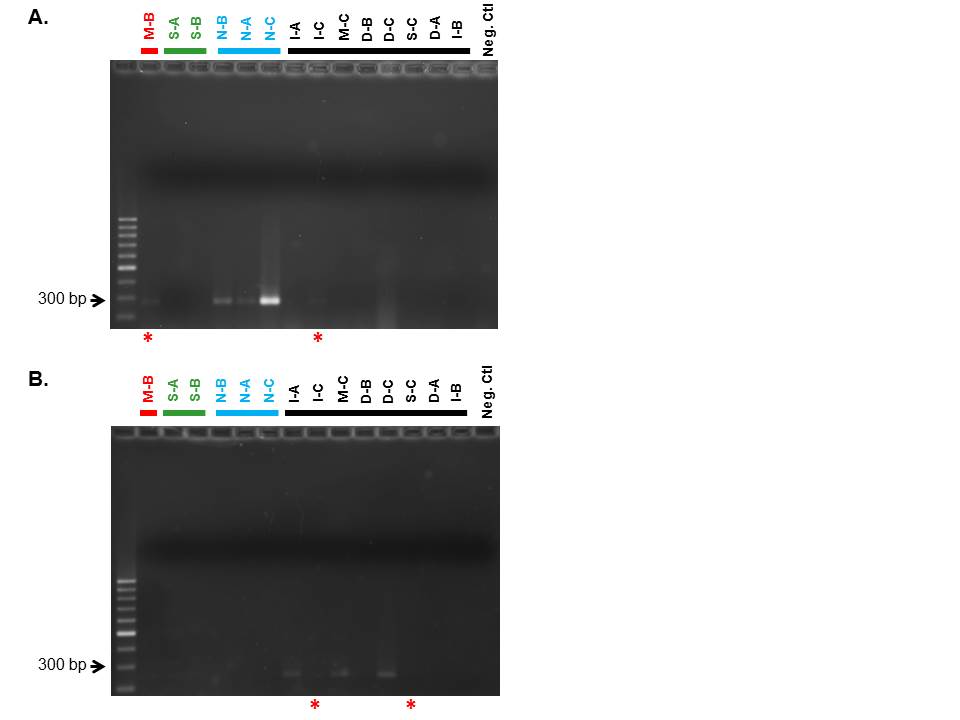


Table S1. Oligonucleotides and PCR conditions utilized to detect bioindicator for baseline soil multifunctional attributes and soil samples that are divergent from the baseline multifunctional attributes. Oligonucleotides are specific to the regions V6 and V8 of 16S rRNA gene in selected OTU. All PCR mixtures consisted of 1X reaction buffer (15 mM MgCl_2_), 0.2 mM deoxynucleotide triphosphates, 10 µM of each primer, 1.25 U Fast-Taq polymerase (Feldan^®^, QC, Canada), 2 µl diluted genomic DNA (1:100) and nuclease-free water to obtain a final volume of 50 µL.

| **Assays** | **Oligonucleotides** | **PCR conditions** |
| --- | --- | --- |
| OTU 398  *(Baseline)* | 398f_(v1)_: 5’-GGAGCTGCCGCGGCGAGAGATC-3’  398r_(v1)_ : 5’-ACGATCTTGCAATCTAGCTTCCCACTGTCACC-3’ | 94°C for 5 min, 40 cycles of amplification (denaturing at 94°C for 45 sec, annealing temperature at 59°C for 45 sec and elongation step of 72°C for 45 sec) and final elongation step at 72°C for 5 min. |
| OTU 3283  *(Different from baseline)* | 3283f_(v3)_: 5’-AGATGAGGGAGTGCCCTTCG-3’  3283r_(v3)_: 5’-CTTCGCGACCCTTTGTACCG-3’ | 94°C for 5 min, 40 cycles of amplification (denaturing at 94°C for 45 sec, annealing temperature at 60°C for 45 sec and elongation step of 72°C for 45 sec) and final elongation step at 72°C for 5 min. |

**References**

Anderson, M.J., Ellingsen, K.E., McArdle, B.H., 2006. Multivariate dispersion as a measure of beta diversity. Ecology Letters 9, 683-693.

Oksanen, J., Blanchet, F., Kindt, R., Legendre, P., Minchin, P., O'Hara, R., Simpson, G., Solymos, P., Henry, M., Stevens, H., Wagner, H., 2012. Vegan: community ecology package. R package version 2.0-4.
